# Managed care and patient ratings of the quality of specialty care among patients with pain or depressive symptoms

**DOI:** 10.1186/1472-6963-7-22

**Published:** 2007-02-16

**Authors:** David Grembowski, David Paschane, Paula Diehr, Wayne Katon, Diane Martin, Donald L Patrick

**Affiliations:** 1 Center for Cost and Outcomes Research, University of Washington, Box 359736, Seattle WA, 98125, USA; 2 Department of Health Services, University of Washington, Box 357660, Seattle WA, 98195, USA; 3 Department of Biostatistics, University of Washington, Box 357232, Seattle, WA 98195, USA; 4 Department of Psychiatry and Behavioral Sciences, University of Washington, Box 356560, Seattle, WA 98195, USA; 5 Department of Geography, University of Washington, Box 353550, Seattle, WA 98195, USA

## Abstract

**Background:**

Managed care efforts to regulate access to specialists and reduce costs may lower quality of care. Few studies have examined whether managed care is associated with patient perceptions of the quality of care provided by physician and non-physician specialists. Aim is to determine whether associations exist between managed care controls and patient ratings of the quality of specialty care among primary care patients with pain and depressive symptoms who received specialty care for those conditions.

**Methods:**

A prospective cohort study design was conducted in the offices of 261 primary physicians in private practice in Seattle in 1997. Patients (N = 17,187) were screened in waiting rooms, yielding a sample of 1,514 patients with pain only, 575 patients with depressive symptoms only, and 761 patients with pain and depressive symptoms. Patients (n = 1,995) completed a 6-month follow-up survey. Of these, 691 patients received specialty care for pain, and 356 patients saw mental health specialists. For each patient, managed care was measured by the intensity of managed care controls in the patient's health plan and primary care office. Quality of specialty care at follow-up was measured by patient rating of care provided by the specialists. Outcomes were pain interference and bothersomeness, Symptom Checklist for Depression, and restricted activity days.

**Results:**

The intensity of managed care controls in health plans and primary care offices was generally not associated with patient ratings of the quality of specialty care. However, pain patients in more-managed primary care offices had lower ratings of the quality of specialty care from physician specialists and ancillary providers.

**Conclusion:**

For primary care patients with pain or depressive symptoms and who see specialists, managed care controls may influence ratings of specialty care for patients with pain but not patients with depressive symptoms.

## Background

As managed care has grown in the U.S., industrialized countries also are adopting elements of managed care but for different reasons, mainly to improve system performance [1,2]. Managed care's historical focus on cost control, however, raises concerns about whether managed care is influencing the quality of care [3,4]. In particular, few studies have examined whether managed care is associated with patient perceptions of the quality of care provided by physician and non-physician specialists.

Previous U.S. studies comparing the quality of care in health maintenance organizations (HMOs) and non-HMOs report mixed evidence, although patient dissatisfaction is consistently greater in HMOs than in non-HMOs [5,6]. Today, HMO vs. non-HMO comparisons are problematic because managed care organizations are managing cost and quality in several ways, and there is a continuum of weak-to-strong strategies across managed care organizations, rather than a sharp dichotomy between HMO vs. non-HMO [7]. One solution is to measure the strategies, or "controls," used by health plans and medical offices to manage care, such as whether the health plan pays for care from a specialist provider only if the patient receives prior approval from the primary physician to see the specialist (known as "gatekeeping"), and if the specialist provider is a member of the health plan's provider network (known as "lock-in") [7,8].

Our literature search revealed few studies of whether more vs. less managed care is associated with patient satisfaction with specialist providers. In a national survey of U.S. adults, Blendon et al constructed a "Quality of Specialist Care Index" from 6-items about respondents' last visit with a medical specialist [9]. Adults in HMOs had significantly lower (worse) index scores than adults in private or Medicare fee-for-service health plans. In particular, adults in HMOs were less likely to report their specialist provider genuinely cared about their situation, and that the amount of time spent with the specialist provider was adequate, compared to adults in fee-for-service plans. In a study of 17,196 enrollees of a large health plan in California, Kerr et al report that dissatisfaction with access to specialty care was an important reason for patient-reported desire to disenroll from a managed care plan, and that dissatisfaction was higher in medical groups that required preauthorization to see specialists [10,11]. These findings suggest that patient satisfaction with specialist providers is lower in more versus less managed MCOs.

The relationship between managed care and patient satisfaction with specialists may depend on the patient's medical condition and provider type, although few studies address this question. In a randomized trial comparing care from psychiatrists, psychologists, social workers, and primary physicians in Britain, patients with depression were more satisfied with care from psychologists and social workers, with social workers receiving the highest scores [12,13]. Although 10-fold differences in contact time existed across provider types, satisfaction scores were not associated with contact time [12]. Another randomized trial in Britain also reports that patients with depression were more satisfied with psychological therapy than usual primary care [14].

For patients with physical health problems, cohort studies comparing patient satisfaction for generalists and specialists report mixed findings. Clinical outcomes for low back pain were similar across provider types, but satisfaction was highest among patients seeing chiropractors [15,16]. Satisfaction scores were similar for rheumatoid arthritis patients seen by generalist and specialist physicians [17]. Satisfaction was generally low-to-moderate for patients with persistent facial pain seeing a wide variety of specialists [18].

Our purpose is to examine the associations between managed care controls and patient perceptions of the quality of specialty care among primary care patients with pain or depressive symptoms who received specialty care for those conditions. For both conditions, the primary null hypothesis is that managed care controls are not associated with ratings of specialty care. We use longitudinal data to control for point-in-time health status and health status change, which may account for any managed care – perceived quality of specialty care relationship [19-22]. Data exist for patients with depressive symptoms to test a secondary hypothesis of whether ratings are similar across provider types.

## Methods

### Design and populations

Data for this analysis come from the Physician Referral Study, a prospective cohort study examining managed care, access to specialists, and outcomes among primary care patients with pain and/or depressive symptoms [23,24]. Written consent was obtained from all patients participating in the study after procedures and possible risks and benefits were explained fully (University of Washington Internal Review Board Application Nos. 96-3204-C and 01-0043-E).

The physician population consisted of 832 primary care physicians (family practitioners, general internists, and general practitioners) in private practice at least 50% time in the Seattle metropolitan area in 1997. Of these, 261 physicians (31%) in 72 offices consented to participate. Participating physicians and their office manager, as well as a random sample of 300 non-participating physicians, were asked to complete self-administered questionnaires at baseline.

In total, 17,187 English-speaking patients aged 18 and over were screened in the waiting rooms of the 72 offices for 2 weeks. Of these, 691 patients were ineligible due to age below 18 or language, physical or mental limitations, and 4,107 eligible patients refused to participate. Of the remaining 12,389 patients, 2,850 consenting patients had elevated depressive symptoms (6 items from the Symptom Checklist for Depression: sleep that is restless or disturbed, feeling low in energy or slowed down, blaming yourself for things, feeling lonely or blue, feeling hopeless about the future, feeling everything is an effort) and/or at least one of eight common, often persistent pain symptoms (back and neck pain, chest pain, abdominal pain, sinus or facial pain, headache or migraine, pain from indigestion/constipation, pain or arthritis in arms/legs/joints, and pelvic pain from female problems) [25]. Three patient cohorts were recruited: (1) patients with pain only (n = 1,514; 53%); (2) patients with pain and depressive symptoms (n = 761;27%); and (3) patients with depressive symptoms only (n = 575; 20%). Patients completed mail or telephone surveys at 1-month and 6-months to collect measures of utilization of specialists, ratings of care from specialists, and health status.

For patients with depressive symptoms, utilization of mental health specialists was defined as patient report of visiting a psychiatrist or other mental health specialist (or both) within 6 months after enrollment. For patients with pain, specialist utilization was defined as seeing a health care professional other than their primary care physician for the pain problem. In the survey instrument for patients with pain, respondents reported whether they visited one or more of the following health care professionals for their pain problem within 6 months after their baseline visit with their primary care physician: acupuncturist, allergist, cardiologist (heart doctor) chiropractor, ear/nose/throat doctor, family doctor or general practitioner, gastroenterologist (digestive system doctor), massage therapist, neurologist, neurosurgeon, obstetrician/gynecologist, orthopedic surgeon, osteopathic physician, physical therapist or occupational therapist, psychiatrist, other mental health practitioner, pulmonary doctor (lung doctor), rehabilitation doctor, rheumatologist (arthritis doctor), other surgeon, or another health care professional reported by the respondent. Based on responses to these items, specialist utilization was defined as patient report of seeing a specialist physician (MD/DO) or other ancillary provider for the pain problem within 6 months after enrollment.

### Patient-rated quality of care measures

If a patient with depressive symptoms reported seeing one or more mental health specialists between the waiting room screen and the 6-month follow-up, the patient rated the care provided by each mental health specialist on a 6-point scale of poor (1), fair, good, very good, excellent, and outstanding (6) [13,26-28]. The type of specialist was coded as psychiatrist vs. other mental health specialist.

If a pain patient reported seeing one or more specialist physicians (MD/DO) or other ancillary providers (such as a physical therapist, massage therapist, chiropractor, or acupuncturist) for the pain problem at the 6-month follow-up, the patient rated the care from the provider(s) on the same 6-point scale. We counted the number of provider types, based on the physician or ancillary provider's specialty, for each patient.

We asked patients to rate the care provided by their primary physician and examined whether ratings for primary and specialty care providers were similar.

### Managed care measures

Based on our conceptual model of managed care [7], we identified managed care controls at two levels of health care organization, health plans and primary care offices, and created indices measuring the intensity of the managed care controls at each level.

#### Health plan indices

For managed care by health plans, we collected information from medical offices and patient screening to identify each patient's source of health insurance (e.g., a health insurance firm, Medicare for adults aged 65 and over, Medicaid for low-income adults), and we collected information for all health plans offered by each source.

A managed care index and three benefit and cost-sharing indices, each ranging from 0-to-100, were constructed using principal component analysis from measures of plan characteristics [8]. A *plan managed care index *(where 100 was a highly managed health plan) measured the intensity of provider-oriented controls in a health plan based on the gatekeeping and lock-in provisions of the plan's network, the plan's referral preauthorization requirements, and whether the plan vs. the provider was at financial risk.

Benefit and cost-sharing indices were developed because some managed plans, such as preferred provider organizations, control costs partly through greater patient cost-sharing. An *in-network benefits index *measured the benefits (services covered) and cost-sharing (co-payments, coinsurance and deductibles) in a plan's network, where 100 indicates the least out-of-pocket cost for standard benefits when services are delivered by providers in the network. An *out-of-network benefits index *measured the benefits and cost-sharing outside a plan's network, where 100 indicates the least out-of-pocket cost for standard benefits when services are delivered by providers outside the plan's network. A *mental health benefits index *measured the inpatient and outpatient mental health benefits inside and outside the plan's network, where 100 indicates the least out-of-pocket costs. The construction and validity of the indices were reported elsewhere [8]. In addition, we measured whether or not the plan had a mental health carve-out [29].

#### Primary care office index

The intensity of managed care in primary care offices was measured through the following controls: utilization management (the office's referral preauthorization requirements), financial incentives (percentage of office revenue from capitation), and whether the office uses referral guidelines or clinical guidelines for specific conditions. Because the office variables were correlated strongly, we created an *office managed care index *using principle component analysis. A single factor explained 60% of the total variation of the five variables. Factor scores were transformed to create a 0–100 office managed care index, where higher scores indicated more managed offices.

### Patient characteristics

Patient measures included age, gender, race, living alone, employment status, education, annual household income. The number of comorbidities at baseline was assessed using a checklist of 21 comorbid conditions based on the Medical Outcomes Study [30]. We also measured the context of care, whether the primary physician at baseline was the patient's usual source of care, whether the baseline visit was the patient's first visit with the primary physician for the pain problem, and whether the patient had sought care for the pain problem in the 6-months before the baseline visit.

For patients with pain, the severity of pain symptoms was measured at the waiting room screen and the 6-month follow-up by a 10-point scale indicating the bothersomeness of the pain in the past 4 weeks, where "0" indicates "not bothersome" and "10" indicates "extremely bothersome" [31]. Functional health status was measured by the 3-item pain interference scale, where "0" indicates "no interference and "10" indicates "unable to carry on activities" [32]. Disability was measured by the number of days the patient was limited in usual activities due to physical health problems in the past 4 weeks [33].

For patients with depressive symptoms, the severity of these symptoms was measured at the waiting room screen and the 6-month follow-up by the 20-item Symptom Checklist for Depression (SCL-20) [34]. Disability was measured by the number of restricted activity days due to emotional problems in the past 4 weeks [33].

For each measure, health outcome was calculated as the change in health status between the waiting room screen and the 6-month follow-up, where bigger values indicated more improvement.

### Data analysis

Patients receiving specialty care between baseline and the 6-month follow-up were identified, and descriptive statistics were computed of all dependent and independent variables for those patients with pain, patients with depressive symptoms, and patients with pain and depressive symptoms. Bivariate statistical tests were performed to determine whether baseline personal characteristics and managed care measures were significantly different across the three cohorts.

Paired *t*-tests were performed to determine whether health status improved between baseline and follow-up. For patients with depressive symptoms, analysis of variance tests were performed to determine whether patient-rated quality of care and health outcomes differed by type of mental health specialist. We also examined these associations separately for patients with depressive symptoms and patients with pain and depressive symptoms. For pain patients, a Pearson correlation estimated the association between the number of specialist types seen for the pain problem and the rating of specialty care.

The relationships between managed care and patient-rated quality of specialty care were examined initially for patients with pain. Four regression models, one for each managed care variable, were estimated to determine the association between each managed care variable and patient ratings of the quality of specialty care for the pain problem. Covariates for both conditions included the patient's age, gender, race, marital status, education, annual household income, employment status, and the number of comorbid conditions. Covariates for patients with pain also included the following baseline health characteristics: pain interference, pain bothersomeness, restricted activity days due to physical health, presence or absence of depressive symptoms, whether the primary care physician was the patient's usual source of care, whether the patient was seeing the primary physician for the first time about the pain problem, and whether the patient reported seeing a health professional for the pain problem in the 6 months prior to the waiting room screen. We reran the models also controlling for whether a patient saw a physician specialist or ancillary provider or both for pain.

Six regression models, one for each managed care variable for patients with depressive symptoms, were estimated to determine the association between each managed care variable and patient ratings of the quality of care from mental health specialists. Additional covariates for patients with depressive symptoms included the following health characteristics at the waiting room screen: SCL depression score, restricted activity days due to emotional health, presence or absence of pain, and whether the primary care physician was the patient's usual source of care. We also reran the models controlling for the type of mental health specialist seen by the patient.

In addition, because health outcomes may influence patient perceptions of care, we reran the models controlling for health status at baseline and health status change. To test whether health status at follow-up was associated with patient ratings, we again reran the regression models controlling for health status at the 6-month follow-up and health status change [19-22,35].

Models were estimated with *STATA®*statistical software [36] using general estimating equations (GEE) to account for correlations among patients in the same medical offices.

## Results

About 95% of the participating physicians and 96% of office managers completed the self-administered questionnaire, and 82% of the non-participating physicians completed their questionnaires. Participating and nonparticipating physicians had similar referral rates, board certification, specialty and racial mix, but participants had a higher percentage of group practice and female physicians who had less years in practice, less office hours per week, and fewer patients aged 65 and over than nonparticipating physicians (*p *< .05).

Complete follow-up data were collected for 2,004 insured patients (1,062 with pain only, 518 patients with pain and depressive symptoms, and 424 patients with depressive symptoms only). Patients with complete data were older and had less pain interference with activities or fewer depressive symptoms than excluded patients without follow-ups. Depressed patients with complete data also were less likely to have seen a psychiatrist in the past than patients without follow-ups.

Of the 1,580 patients with pain only or pain and depression, 44% (n = 691) saw one or more specialist physicians or ancillary providers for pain by the 6-month follow-up. Of these, 64% saw a specialist physician, and 59% saw an ancillary provider. On average, these patients saw 1.6 (st dev, 0.86) different types of physician specialists or ancillary providers for their pain problems. Of the 942 patients with depressive symptoms with or without pain, 38% (n = 356) saw one or more mental health specialists. Of these, 54% saw a psychologist or master-level therapist, 12% saw a psychiatrist, and 34% saw both. On average, primary care patients who saw mental health specialists had more severe SCL depression scores at baseline than patients who received only primary care.

Table 1 presents baseline characteristics of patients receiving specialty care. The average ages of patients ranged 42–51 across cohorts. A majority of patients were female, white, married, had education beyond high school, had moderate family incomes, and were seeing their usual primary care physician at the waiting room screen. Patients averaged between 2–3 comorbidities across cohorts. For patients with pain, musculoskeletal pains were the most common. Less than half of the patients were seeing their primary care physician the first time for their pain symptom, and over half had seen a health professional for their pain problem in the past 6 months. For patients with depressive symptoms only, a majority had seen a mental health specialist in the past 6 months before the waiting room screen, but for patients with depressive symptoms and pain, only a minority had done so.

**Table 1:** Baseline Characteristics of Patients Receiving Specialty Care in 6-Month Follow-up Period

**Measure**	**Patients with Pain Seeing Specialists for Pain (n = 466)**	**Patients with Depressive Symptoms Seeing Mental Health Specialists (n = 184)**	**Patients with Pain and Depressive Symptoms Seeing Mental Health Specialists or Specialists for Pain (n = 322)**	***p *value**
**Patient Characteristics**				
Age	51 (16.0)	42 (12.9)	45 (13.9)	<.001
Female	63%	74%	76%	<.001
Nonwhite	9%	9%	14%	.029
Living alone	31%	49%	41%	<.001
Employed	63%	68%	64%	.511
Education (years)	15 (2.5)	14 (2.3)	14 (2.5)	.001
Annual household income	$52,159 (28,291)	$38,206 (25,523)	$40,575 (27,967)	<.001
Number comorbidities	2.5 (1.8)	2.1 (1.8)	3.0 (2.1)	<.001
MD at waiting room screen is patient's usual source of medical care	81%	83%	84%	.481
**Baseline Pain Symptoms (percent)**				
Joint, arm, or leg pain	40		31	.020
Back pain	29		30	.719
Sinus, ear, or facial pain	8		10	.192
Abdominal pain	9		7	.441
Chest pain	5		4	.628
Headache and migraine	6		12	.002
Pain from indigestion and constipation	3		2	.139
Pelvic pain	2		4	.082
**Baseline Pain**				
Pain interference	4.7 (2.9)		6.2 (2.6)	<.001
Pain bothersomeness	6.7 (2.7)		7.5 (2.3)	<.001
Restricted activity days due to physical health	4.8 (7.6)		10.0 (10.3)	<.001
Patients seeing primary physician first time for pain problem at baseline visit	46%		33%	.001
Patients with visits to any health professional for pain problem in 6 moths before baseline visit	60%		79%	<.001
**Baseline Depression**				
SCL Depression Score		2.0 (.7)	1.7 (.6)	<.001
Restricted activity days due to emotional health		8.0 (8.9)	7.1 (8.9)	.262
Patients with visits to a mental health specialist in past 6-months		65%	38%	<.001

On average, pain patients rated the care from specialist physicians and ancillary providers as "very good" (unadjusted avg: 4.3; st dev, 1.2) at the 6-month follow-up. Ratings were similar for patients who saw a single specialist and patients seeing providers with different specialties. Patient ratings of care from primary physicians and specialists were correlated moderately (*r *= .36, *p *= .000). For depression patients seeing psychiatrists, the average rating of their psychiatric care was 3.9 (st dev 1.5). Patients seeing other types of mental health specialists had higher ratings of their mental health care (avg: 4.3; st dev 1.5; *p *< .02). For depression patients, ratings of care delivered by their primary physicians had low correlations with ratings of care from psychiatrists (*r *= .18, *p *= .04) and other mental health specialists (*r *= .22, *p *= .001).

Table 2 describes the health status of patients at the waiting room screen and the 6-month follow-up. On average, most patients improved. Health status scores declined by about half between the waiting room screen and the 6-month follow-up, but restricted activity days due to physical health declined only by 27% for patients with pain and depressive symptoms.

**Table 2:** Health Status at Waiting Room Screen and 6-Month Follow-Up: Unadjusted Descriptive Statistics and Change Scores

	**Patients with Pain Seeing Specialists for Pain**	**Patients with Depressive Symptoms Seeing Mental Health Specialists**	**Patients with Pain and Depressive Symptoms Seeing Mental Health or Pain Specialists**
Depression Outcomes (Averages)		(n = 184)	(n = 317–321)
SCL Depression Score			
Waiting room screen		2.0	1.7
6-Month follow-up		1.1	1.0
Change score**		0.9*	0.7*
Restricted Activity Days Due to Emotional Health			
Waiting room screen		8.0	6.9
6-Month follow-up		4.2	3.5
Change score		3.8*	3.4*
			
Pain Outcomes (Averages)	(n = 416–459)		(n = 318–319)
Pain Interference (n = 459)			
Waiting room screen	4.7		6.2
6-Month follow-up	2.1		3.4
Change score	2.6*		2.8*
Pain Bothersomeness (n = 458)			
Waiting room screen	6.7		7.5
6-Month follow-up	3.0		4.1
Change score	3.7*		3.4*
Restricted Activity Days Due to Physical Health (n = 416)			
Waiting room screen	4.8		10.0
6-Month follow-up	2.6		7.3
Change score	2.2*		2.7*

* Difference between averages is significant (*p *< .001)

** Difference in SCL change scores between the patients with depressive symptoms only vs. patients with pain and depressive symptoms is significant (*p *= .002). All other cohort comparisons of change scores are not statistically significant (*p *> .05).

**Figure 1:**
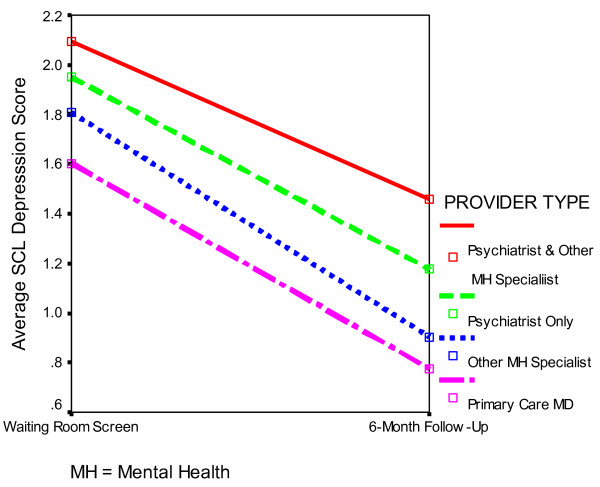
Change in SCL Depression Scores between Waiting Room Screen and 6-Month Follow-up by Type of Provider(s) Seen.

Figure 1 illustrates average SCL depression scores at the waiting room screen and the 6-month follow-up for patients seeing different types of providers. Unadjusted average SCL change scores by provider type were as follows: primary physicians only, 0.83; other mental health specialists, 0.90; psychiatrists only, 0.77; psychiatrists and other mental health specialists, .63; *p *= .014).

For patients with depressive symptoms only, average SCL change scores were not significantly different for patients seeing psychiatrists only (.74), other mental health specialists only (1.00), and both psychiatrists and other mental health specialists (.86; *p *= .32). For patients with pain and depressive symptoms, average SCL change scores were significantly different for patients seeing psychiatrists only (.80), other mental health specialists only (.78), and both psychiatrists and other mental health specialists (.42; *p *= .02). For pain patients, seeing a greater number of providers with different specialties was not associated with greater improvement.

Table 3 presents descriptive statistics of the health plan and office managed care variables for patients seeing specialists. The correlation between the health plan managed care index and the office managed care index was .38 (*p *= .000).

**Table 3:** Descriptive Statistics of Managed Care Indices for Health Plans and Primary Care Offices

**Managed Care Measures**	**Patients with Pain Seeing Specialists**	**Patients with Depressive Symptoms Seeing Mental Health Specialists**	**Patients with Pain and Depressive Symptoms Seeing Mental Health or Pain Specialists**	***p *value**
Health Plan Indices (Averages)	(n = 383)	(n = 149)	(n = 269)	
Plan Managed Care Index	39	38	34	.069
In-Network Benefits Index	90	90	91	.021
Out-of-Network Benefits Index	43	45	47	.455
Mental Health Benefits Index	--	52	57	.096
Mental Health Carve Out (%)	--	26%	30%	.313
				
Office Managed Care Index (Averages)	(n = 434)	(n = 171)	(n = 322)	
Office Managed Care Index^1^	43	37	37	.026

^1^Patients were seen in offices that, on average, received 28% of their revenue from capitation. About 20% of patients were seen in offices where primary physicians required prior approval to refer inside the practice; about 62% of the patients were seen in offices where prior approval was required to refer outside the practice; and about 38% of the patients were seen in offices with guidelines.

### Managed care and quality of specialty care

For patients with pain and depressive symptoms, the plan managed care index, the benefit indices, and the mental health carve out variable were not associated with patient-rated quality of specialty care in all regression models.

In contrast, pain patients in more managed primary care offices had lower ratings of the quality of care from physician and non-physician specialists (coefficient of office managed care index: -.004; *p *= .004). The same result was obtained in regression models that controlled for whether the patient saw a physician or non-physician specialist, and in models that controlled for change in health status. The pain measures at baseline and 6-month follow-up, as well as change scores, were generally not statistically significant and had inconsistent associations across regression models. However, patients who had more restricted activity days at baseline (*p *= .03), or who had a greater reduction in restricted activity days (*p *= .04), had higher ratings of care provided by physician and non-physician specialists.

For patients with depressive symptoms, the office managed care index was not associated with patient-rated care from mental health specialists in all regression models. More severe SCL depression scores at either baseline or the 6-month follow-up were associated consistently with lower ratings of mental health specialists in regression models, but no association existed between SCL change scores and patient-rated care from mental health specialists.

## Discussion

For primary care patients with pain or depressive symptoms who received specialty care, we examined whether the intensity of managed care controls in their health plans or primary care offices were associated with their ratings of specialty care. We generally found that the intensity of managed care controls in health plans and primary care offices was not associated with patient ratings of the quality of specialty care. However, pain patients in more managed primary care offices had lower ratings of the quality of specialty care from physician specialists and ancillary providers. This association is consistent with Blendon et al [9] and our previous results that pain patients in more managed offices had lower ratings of care provided by their primary physicians [24].

We examined whether these associations might be explained by patient health status, which previous studies had shown to be related to patient ratings of care. When we controlled for health status at baseline, or health status at the 6-month follow-up, or for change in health status, the same results were obtained.

For patients with depressive symptoms, the lack of associations between managed care controls and ratings of care from mental health specialists may have occurred for several reasons. First, the sample of patients seeing mental health specialists was relatively small, and power may be inadequate to detect smaller differences between patients in low vs. high managed settings.

Second, patients with more severe depression symptoms are more likely to see psychiatrists and to rate their psychiatric care lower, which reflects the inverse correlation between severity of depression and patient ratings. However, patients in more managed plans are less likely to see psychiatrists [23], which may have negated potential associations between managed care controls and ratings of mental health care.

Third, we did not measure managed care controls targeting specialists, such as limits on the number of specialist visits and how specialists were paid. In some cases, managed care may increase access to mental health specialists but limit the number of visits. The intensity of managed care controls facing specialists may be related to patient perceptions of the quality of specialty care.

Last, patients may have developed close relationships with their mental health specialists, and consequently, patient ratings of mental health care may be influenced more by patient-specialist relationships than by managed care controls. In contrast, pain patients, who generally seek pain relief, may not have developed similar, close relationships with their specialists, and without this patient-specialist relationship "buffer," managed care controls in primary offices may contribute to their lower ratings of specialty care.

Our findings have policy implications for improving the quality of mental health care. The United States, the United Kingdom and other countries are exploring pay-for-performance (P4P) incentives to improve the quality of care for physical and mental health problems [37]. One type of P4P is paying specialty providers based on patient ratings of their care [38]. Our findings provide pilot information about the levels and variation in ratings of specialty providers for patients with pain or depressive symptoms, but little is known about whether P4P programs can influence such ratings.

Our patient ratings of care from mental health specialists indicate that room for improvement exists, and developing interventions to improve the overall quality of mental health care is an important priority [38]. Collaborative care for primary care patients is one mechanism for improving outcomes and ratings of mental health care among primary care patients with depressive symptoms [39,40]. Our findings suggest that the presence or absence of managed care controls that we examined may not influence the performance of such interventions.

### Limitations

Our findings are limited to our sample of mainly middle income, Caucasian adults with pain or depressive symptoms in the private practices of consenting family practitioners, general internists, and general practitioners in the Seattle area. Primary physicians in small practices were less likely to participate, and our findings may not apply to patients in those settings.

The Seattle patients had a relatively even distribution of traditional indemnity health plans, preferred provider organizations, point of service plans, and health maintenance organizations, and were seen in a variety of primary care organizations, ranging from solo practice to integrated delivery systems. Our findings may not be generalizable to other cities with different mixes of managed care and delivery systems.

Another limitation of observational studies is that patients and physicians are not randomized to health plans and medical offices, so our results may be influenced by selection bias.

## Conclusion

For primary care patients with pain or depressive symptoms who received specialty care, the intensity of managed care controls in health plans and primary care offices was generally not associated with patient ratings of the quality of specialty care. However, pain patients in more managed primary care offices had lower ratings of the quality of specialty care from physician specialists and ancillary providers.

## Competing interests

The author(s) declare that they have no competing interests.

## Authors' contributions

DG, principal investigator, was responsible for the conduct of the overall study and primary author. DP, research assistant, was responsible for data analyses performed under the direction of the investigators and producing results tables and documentation of data analysis protocols and results. PD, biostatistician, developed managed care indices with the principal investigator, guided data analyses, and interpreted results. WK assisted in overall study design, developing data collection protocols in medical offices, selection of measures of depression, and interpretation of results. DM assisted in designing data collection protocols, selection of measures of provider satisfaction and managed care, and interpretation of results. DP assisted in designing data collection protocols, selection of measures of pain and disability, and interpretation of results. All authors read and approved the final manuscript.

## Pre-publication history

The pre-publication history for this paper can be accessed here:



## References

[B1] Donaldson C, Ruta D (2005). Should the NHS follow the American way?. BMJ.

[B2] Grembowski DE, Cook KS, Patrick DL, Roussel AE (2002). Managed care and the U.S. health care system: A social exchange perspective. Social Science & Medicine.

[B3] Light DW, Albrecht GL, Fitzpatrick R, Scrimshaw SC (2000). The sociological character of health-care markets. The Handbook of Social Studies in Health and Medicine.

[B4] Mechanic D, Rochefort DA (1996). Comparative medical systems. Ann Rev Sociol.

[B5] Miller RH, Luft HS (2002). HMO plan performance update: an analysis of the literature, 1997–2001. Health Affairs.

[B6] Dudley RA, Miller RH, Korenbrot TY, Luft HS (1998). The impact of financial incentives on quality of care. Milbank Quarterly.

[B7] Grembowski DE, Cook K, Patrick DL, Roussel AE (1998). Managed care and physician referral. Medical Care Research and Review.

[B8] Grembowski DE, Diehr P, Novak LC, Roussel AE, Martin DP, Patrick DL, Williams B, Ulrich CM (2000). Measuring the managedness and covered benefits of health plans. Health Services Research.

[B9] Blendon RJ, Knox RA, Brodie M, Benson JM, Chervinsky G (1994). Americans compare managed care, Medicare, and fee-for-service. Journal American Health Policy.

[B10] Kerr EA, Hays RD, Lee ML, Siu AL (1998). Does dissatisfaction with access to specialists affect the desire to leave a managed care plan?. Medical Care Research and Review.

[B11] Kerr EA, Hays RD, Mitchinson A, Lee M, Siu AL (1999). The influence of gatekeeping and utilization review on patient satisfaction. J Gen Intern Med.

[B12] Scott AI, Freeman CP (1992). Edinburgh primary care depression study: treatment outcome, patient satisfaction, and cost after 16 weeks. BMJ.

[B13] Scott J, Moon C, Blacker C, Thomas JM (1994). A. I. F. Scott & C.P.L. Freeman's Edinburgh Primary care depression study. British Journal of Psychiatry.

[B14] Ward E, King M, Lloyd M, Bower P, Sibbald B, Farrelly S, Gabbay M, Tarrier N, Addington-Hall J (2000). Randomised controlled trial of non-directive counseling, cognitive-behavioral therapy, and usual general practitioner care for patients with depression. I: Clinical effectiveness. BMJ.

[B15] Solomon DH, Bates DW, Panush RS, Katz JN (1997). Costs, outcomes, and patient satisfaction by provider type for patients with rheumatic and musculoskeletal conditions: a critical review of the literature and proposed methodological standards. Annals of Internal Medicine.

[B16] Nyiendo J, Haas M, Goldberg B, Sexton G (2001). Pain, disability, and satisfaction outcomes and predictors of outcomes: a practice-based study of chronic low back pain patients attending primary care and chiropractic physicians. J Manipulative Physiol Ther.

[B17] Bidaut-Russell M, Gabriel SE, Scott CG, Zinsmeister AR, Luthra HS, Yawn B (2002). Determinants of patient satisfaction in chronic illness. Arthritis & Rheumatism.

[B18] Turp JC, Kowalski CJ, Stohler CS (1998). Treatment-seeking patterns of facial pain patients: many possibilities, limited satisfaction. Journal of Orofacial Pain.

[B19] Patrick DL, Scrivens E, Charlton J (1983). Disability and patient satisfaction with medical care. Medical Care.

[B20] Marshall GN, Hays RD, Mazel R (1996). Health status and satisfaction with health care: results from the Medical Outcomes Study. J Consult Clin Psychol.

[B21] Kane RL, Maciejewski M, Finch M (1997). The relationship of patient satisfaction with care and clinical outcomes. Medical Care.

[B22] Covinsky KE, Rosenthal GE, Chren MM, Justice AC, Fortinsky RH, Palmer RM, Landefeld CS (1998). The relation between health status changes and patient satisfaction in older hospitalized medical patients. Journal of General Internal Medicine.

[B23] Grembowski DE, Martin D, Patrick D, Diehr P, Katon W, Williams B, Engelberg R, Novak L, Dickstein D, Deyo R, Goldberg HI (2002). Managed care, access to mental health specialists, and outcomes among primary care patients with depressive symptoms. Journal of General Internal Medicine.

[B24] Grembowski DE, Martin D, Diehr P, Patrick DL, Williams B, Novak L, Deyo R, Katon W, Dickstein D, Engelberg R, Goldberg H (2003). Managed care, access to specialists, and outcomes among primary care patients with pain. Health Services Research.

[B25] National Centers for Health Statistics (1992). National ambulatory medical care survey: 1989 summary. Vital and Health Statistics Series.

[B26] Hays RD, Shaul JA, Williams VS, Lubalin JS, Harris-Kojetin LD, Sweeny SF, Cleary PD (1999). Psychometric properties of the CAHPS^® ^1.0 survey measures. Consumer Assessment of Health Plans Study. Med Care.

[B27] Ware JE, Hays RD (1988). Methods for measuring patient satisfaction with specific medical encounters. Medical Care.

[B28] Grembowski D, Patrick DL, Williams B, Diehr P, Martin DP (2005). Managed care and patient-rated quality of care from primary physicians. Med Care Res Rev.

[B29] Frank RG, Huskamp HA, McGuire TG, Newhouse JP (1996). Some economics of mental health 'carve-outs'. Arch Gen Psychiatry.

[B30] Wells K, Rogers W, Burnam A, Greenfield S, Ware JE (1991). How the medical comorbidity of depressed patients differs across health care settings: results from the Medical Outcomes study. American Journal of Psychiatry.

[B31] Patrick DL, Deyo RA, Atlas SJ, Singer DE, Chapin A, Keller RB (1995). Assessing health-related quality of life in patients with sciatica. Spine.

[B32] Von Korff MJ, Ormel FJ, Keefe SF, Dworkin SF (1992). Grading the severity of chronic pain. Pain.

[B33] Ware J, Kosinski MS, Keller D (1996). A 12-item short-form health survey: construction of scales and preliminary tests of reliability and validity. Medical Care.

[B34] Derogatis LR, Lipman RS, Rickels K, Uhlenhuth EH, Covi L, Pichot P (1974). The Hopkins Symptom Checklist: a measure of primary symptom dimensions. Psychological measurements in psychopharmacology: Problems in pharmacopsychiatry.

[B35] Kaplan SH, Greenfield S, Ware JE (1989). Assessing the effects of physician-patient interactions on the outcomes of chronic disease. Medical Care.

[B36] Stata Corporation (1999). STATA: statistics/data analysis.

[B37] Lester H (2006). Current issues in providing primary medical care to people with serious mental illness. Int J Psychiatry Med.

[B38] Patel KK, Butler B, Wells KB (2006). What is necessary to transform the quality of mental health care?. Health Affairs.

[B39] Katon W, Von Korff M, Lin E, Simon G, Walker E, Unutzer J, Bush T, Russo J, Ludman E (1999). Stepped collaborative care for primary care patients with persistent symptoms of depression: a randomized trial. Arch Gen Psychiatry.

[B40] Katon W, Unutzer J (2006). Collaborative care models for depression: time to move from evidence to practice. Arch Intern Med.

